# Personalization of cancer treatment using predictive simulation

**DOI:** 10.1186/s12967-015-0399-y

**Published:** 2015-02-01

**Authors:** Nicole A Doudican, Ansu Kumar, Neeraj Kumar Singh, Prashant R Nair, Deepak A Lala, Kabya Basu, Anay A Talawdekar, Zeba Sultana, Krishna Kumar Tiwari, Anuj Tyagi, Taher Abbasi, Shireen Vali, Ravi Vij, Mark Fiala, Justin King, MaryAnn Perle, Amitabha Mazumder

**Affiliations:** New York University School of Medicine, New York, NY USA; Cellworks Research India Pvt. Ltd, Bangalore, India; Cellworks Group, Inc, San Jose, CA USA; Washington University School of Medicine, St. Louis, MO USA; New York University Cancer Center, New York, NY USA

**Keywords:** Multiple myeloma, Rational drug design, Personalized therapy

## Abstract

**Background:**

The personalization of cancer treatments implies the reconsideration of a one-size-fits-all paradigm. This move has spawned increased use of next generation sequencing to understand mutations and copy number aberrations in cancer cells. Initial personalization successes have been primarily driven by drugs targeting one patient-specific oncogene (e.g., Gleevec, Xalkori, Herceptin). Unfortunately, most cancers include a multitude of aberrations, and the overall impact on cancer signaling and metabolic networks cannot be easily nullified by a single drug.

**Methods:**

We used a novel predictive simulation approach to create an avatar of patient cancer cells using point mutations and copy number aberration data. Simulation avatars of myeloma patients were functionally screened using various molecularly targeted drugs both individually and in combination to identify drugs that are efficacious and synergistic. Repurposing of drugs that are FDA-approved or under clinical study with validated clinical safety and pharmacokinetic data can provide a rapid translational path to the clinic. High-risk multiple myeloma patients were modeled, and the simulation predictions were assessed *ex vivo* using patient cells.

**Results:**

Here, we present an approach to address the key challenge of interpreting patient profiling genomic signatures into actionable clinical insights to make the personalization of cancer therapy a practical reality. Through the rational design of personalized treatments, our approach also targets multiple patient-relevant pathways to address the emergence of single therapy resistance. Our predictive platform identified drug regimens for four high-risk multiple myeloma patients. The predicted regimes were found to be effective in ex vivo analyses using patient cells.

**Conclusions:**

These multiple validations confirm this approach and methodology for the use of big data to create personalized therapeutics using predictive simulation approaches.

**Electronic supplementary material:**

The online version of this article (doi:10.1186/s12967-015-0399-y) contains supplementary material, which is available to authorized users.

## Background

Multiple myeloma (MM) is a blood disorder that impairs and suppresses the immune system [1,2]. MM is the second most common hematological malignancy and is characterized by the infiltration, expansion and survival of malignant plasma cells in the bone marrow that typically become resistant to all chemotherapies. The development of drug resistance is a significant clinical obstacle in the treatment of MM. High levels of intra-patient heterogeneity in malignant cells in terms of genetic aberrations and characteristics as well as interactions with the bone marrow microenvironment are the major determinants of MM resistance mechanisms [3-7]. Despite a similar manifestation of disease endpoints, inter-patient heterogeneity in cellular profiles results in a wide variety of responses to similar drugs among patients. In addition, the lack of a common recurrent mutation provides further reason to explore this disease on a personalized basis. This presents an opportunity to develop and design personalized therapies based on the specific set of cellular profiles and microenvironment influences that can also differ between patients.

The well-defined standard of care treatments currently used for MM include proteasome inhibitors, such as bortezomib and carfilzomib, and immunomodulator (IMiD) drugs, including thalidomide, linalidomide and pomalidomide. Conventional cytotoxic chemotherapy drugs, such as melphalan, cyclophosphamide, and doxorubicin, are also used in MM therapy. Stem cell transplantation is another treatment option available for MM [3]. MM patients typically initially respond to the primary standard of care, and then a few develop resistance or simply do not respond to the existing and available standards of treatment. This lack of response could be attributed to the presence of CD138^+^ and CD138^−^ clones in patients, which differentially respond to drug therapies [4]. Over time, the sensitive clone is eliminated, and the resistant clone subsequently dominates, resulting in disease recurrence. Additionally, factors and conditions in the bone marrow microenvironment, including increased levels of cytokines and chemokines (IL6 and CCL5), stem cell signaling mediated by WNT and NOTCH, and hypoxic microenvironments, also contribute to drug resistance [8-13].

**Figure 1:**
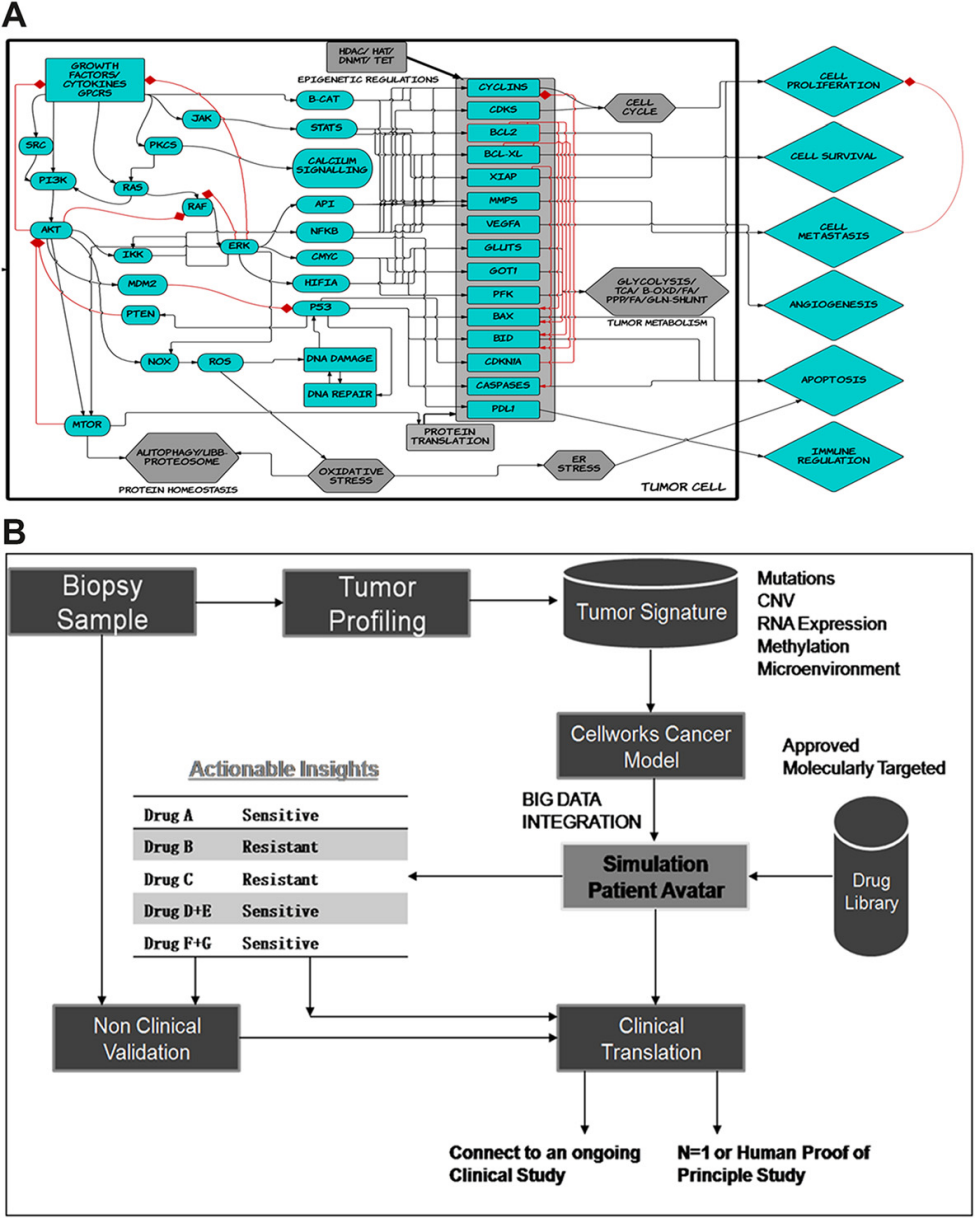
**Predictive platform design and study workflow. (A)** Schematic representing the crosstalk between various pathways in the plasma cell simulation platform. An oversimplified graphical representation of the signaling components, including key kinases, transcription factors and genes, that underlie various cancer phenotypes incorporated in the network is presented. The schematic also highlights cellular processes that are modeled, including epigenetic regulation, tumor metabolism, oxidative stress, protein homeostasis (proteasome and autophagy), cell cycle machinery and DNA repair pathways. The crosstalk between all these pathways represents our control non-triggered simulation platform that can be transitioned into the respective disease state by triggering the mutations and aberrations reported for a profile. **(B)** Description of process for designing personalized therapy: The process flow for the N = 1 personalized therapy design as described in the Methods section is presented. A bone marrow sample from the patient tumor is profiled, and the tumor signature consisting of CNV, mutations and other relevant data is input into the simulation model to create the simulation patient avatar. A drug library is tested on this patient avatar to identify drugs and therapies predicted to be sensitive or resistant in the particular profile. This information can be validated *ex viv*o via non-clinical validation or clinically translated by either connecting to a specific ongoing clinical study or the personalized N = 1 option for that particular patient.

In this study, we used predictive simulation modeling of cancer physiology to design and shortlist therapies predicted to overcome MM resistance. Cancer physiology simulation technology (Cellworks Group, San Jose, CA, USA) can be used to conduct high-throughput studies to assess complex biological mechanisms resulting from drug treatment [14-18]. Specifically, the system predicts mechanisms using drug combinations that synergistically interact to reduce viability, proliferation and other biologically relevant endpoints. The predictive simulation technology comprehensively incorporates integrated networks of signaling and metabolic pathways that underlie all cancer phenotypes [19]. A high-level schematic of the network circuitry of key signaling pathways, message transduction cascades and transcription factor-mediated regulation of gene expression along with cellular processes incorporated into the plasma cell simulation platform is presented in Figure 1A. A list of abbreviations that appear in the pathway schematic can be found in Additional file 1. Using genomic profiling information from patient tumors as inputs into the system, a patient simulation avatar is created. This predictive platform was then used to test a library of molecularly targeted drugs on the patient simulation avatar. Drug agents were combined at different dose ratios via simulation-based studies, resulting in analyses of the effects of a large number of drug combinations on functional phenotypes, including proliferation, viability, angiogenesis and biomarker expression. The shortlisted novel combinations were experimentally validated *ex vivo* in corresponding patient cells at the phenotype and biomarker levels. Our predictions were confirmed by our experimental results.

The predictive simulation approach differs from other pathway modeling approaches. Unlike a static network, big data mining or informatics approach, this approach enables novel drug simulations and predictions prospectively validated using clinical and biomarker endpoints. The integrated cancer physiology network that covers all disease phenotypes in the simulation model are aggregated through manual scientific review one reaction at a time to maintain a high quality of input information and address issues of prevalent contradictory datasets. In addition, the network is continuously enhanced with information from new research. The semiconductor engineering methodology and infrastructure makes feasible a large quantum of simulation dose response studies to shortlist specific therapeutic and biomarker assets from millions of studies.

## Methods

### Collection of samples and growing patient cell line

Samples were obtained after informed consent was provided by patients in accordance with the Human Subjects Protection Committee and the Declaration of Helsinki. Human experimentation guidelines of the United States Department of Health and Human Services and those of New York University were followed. This study was approved by the New York University Institutional Review Board (protocol number 06-523) and the Washington University School of Medicine Institutional Review Board (HRPO 201102270). All of the patients selected for this study failed all the existing approved standard of care treatments for myeloma. Bone marrow aspirates were collected in heparinized tubes and then diluted 1:1 with Leibovitz L-15 medium (Life Technologies) supplemented with fetal bovine serum, interleukin [IL]-6 (IL-6), vascular endothelial growth factor (VEGF), insulin-like growth factor-1 (IGF-1).The mononuclear cell fraction was enriched by Ficoll-Hypaque (Pharmacia) density gradient sedimentation at 600 g for 20 min and washed twice in L-15/fetal bovine serum media as previously described [20]. For sample 5, CD138^*+*^ plasma cell isolation was performed by FACS analysis. Cells were then propagated in RPMI media supplemented with IL-6, VEGF and IGF-1. Cells were co-cultured with the support of M210B4 stromal cells (Cases 1-3) or patient stromal cells (Case 4) [21].

### Genomic signature of patient myeloma cells

Results from genome-wide assays that report deviations from the genome of a non-cancerous cell were used to provide information about the patient’s genomic signature. We used data from aCGH (array comparative genomic hybridization) and cytogenetic profiling to create the simulation avatar of MM patients. aCGH is a molecular cytogenetic method for analyzing copy number variations (CNV) relative to DNA ploidy levels in a test sample compared with a reference sample, such as malignant tumor cells compared with normal cells of the same patient. aCGH can detect aneuploidies, including deletions, duplications, and/or amplifications, of any locus represented on an array, thus providing a snapshot of the patient’s tumor genomic signature. CD138- cells from the bone marrow sample were used as the non-cancerous reference for each patient. Cytogenetic profiling by spectral karyotyping (SKY) reports chromosomal aberrations, including loss/gain of complete chromosomes or specific chromosomal regions resulting in monosomy/trisomy of the genes in the affected region. In addition to deletions and duplications, other abnormalities that are reported include derivative chromosomes, isochromosomes, and translocations. Both aCGH/SKY methods can be further substantiated by FISH (fluorescent *in situ* hybridization) using marker genes in the affected chromosomal regions [22-25]. Additionally, whole genome sequencing (WGS) or exome sequencing data can report CNV as well as point mutations that provide the genomic signature of the patient tumor and enable the creation of the patient simulation avatar.

### Creation of patient simulation avatars

The input genomic data from aCGH/SKY/WGS were used to create a list of all numerical and/or structural aberrations in the patient’s genome. The genes found on the loci of these affected regions of the chromosome are extracted from the human reference genome at ENSEMBL. The complete list of genes is matched with the Cellworks MM model to identify genes that are represented in the model. All genes that have coverage in the model form the trigger file that is used to create the patient’s simulation avatar as previously reported [19]. The genes reported to have gain in copy number are overexpressed, whereas genes with a loss in copy number are knocked-down.

### Simulation protocol

The dynamic cancer model is simulated for 50,000 seconds until the system reaches a homeostatic steady state, thus being initialized to a control state aligned to normal plasma cell physiology that is non-tumorigenic. This non-transformed plasma cell is triggered to transition into a neoplastic state by introducing the patient trigger file. The model is further simulated for 50,000 to 100,000 seconds after the introduction of the triggers that modulate the functional levels of intracellular molecules to align the system to the network dynamics of the patient’s tumor and to achieve a disease steady state. It is important to note that the time to achieve steady state of the disease network varies based on the complexity of the profile definition.

### Designing individualized therapy

The creation and simulation of the patient simulation avatar is followed by high throughput data and network analysis of the system to provide insight into the dominant pathways and signaling loops in the particular patient profile. The patient simulation network is not dominated by one gene mutation or perturbation with a significant phenotypic impact, rather the cumulative effects of the multitude of chromosomal aberrations derived from the genomic datasets that shift the dynamics of the signaling network towards the disease phenotypes of enhanced proliferation, increased viability and reduced apoptosis. Each patient profile is unique with regard to the set of aberrations present, thereby resulting in a significantly unique network configuration. We also model and simulate different clones within a patient sample with slightly different genomic aberrations to incorporate disease heterogeneity. The key characteristics of the patient simulation avatar are represented as a network schematic, including the list of tumor suppressor and tumor promoter genes derived from the reported aberrations to highlight the key pathways and markers that are dominant in the particular profile.

Once the patient simulation avatar is created, a library of drug agents is simulated on the profile for an additional 50,000 to 100,000 seconds. The drug library is tested at physiologically achievable concentrations that will manipulate the target moderately by 50-60%. The drug concentration used in the simulation study is assumed to represent a physiological concentration after drug absorption, distribution, transport, and metabolism. High throughput studies of the drug library are performed on the patient clones at physiological concentrations, and those doses exhibiting a greater than 30% efficacy on the disease endpoints of reduction in tumor relative growth index are shortlisted for further analysis. The tumor relative growth index is a complex predictive phenotype index that includes weighted functions of biomarkers impacting proliferation, survival, and apoptosis, including cell cycle checkpoint CDK-cyclin complexes; pro-survival markers, such as BCL2, MCL1, and BIRC3; and pro-apoptotic markers, such as caspases, Puma, and cleaved PARP1.

Given that small molecule inhibitors are primarily used in the study, the optimal manipulation of the target achievable at physiological drug doses is assumed to be approximately 50% with the exception of biologic drugs, such as antibodies, where the target inhibition would be greater than 90%. The effect of the target manipulation is assessed across a dose range, and the dose used in the combination regimen was chosen based on the percentage of target inhibition or the concentration that results a specific inhibition (inhibitory concentration (IC)20 or IC30) or relative growth.

The mechanisms of action of the shortlisted drugs are analyzed in terms of the patient network dominance, and those individual or combination agents that align with reducing the key biomarkers and converging nodes in the network are selected for experimental *ex vivo* validation using patient tumor cells. For combination therapy, combinations resulting in a synergistic reduction of the biomarkers and endpoints are selected such that the effect is not dominated by one agent. This allows very low concentrations of the drug agents (IC20) to be combined, thereby achieving a greater than IC50 efficacy.

Figure 1B summarizes the process flow for the N = 1 personalized therapy design as described above. Bone marrow samples from the patient are profiled, and the tumor signature comprising of CNV, mutations and other relevant data are input into the simulation model to create the simulation patient avatar. A drug library is tested on this patient avatar to identify the drugs that would be sensitive or resistant in the particular profile. This information is validated *ex vivo* for non-clinical validation and also translated clinically by either connecting to a specific ongoing clinical study or a personalized N = 1 option for that particular patient.

Ex vivo validations use a drug concentration range individually based on drug affinity information available from existing studies. From this dose response study, the IC20 relative growth concentration is determined for each drug in the combination. This concentration is used to assess the efficacy of the drug combination.

Given that most of the drugs in the library are either approved or in clinical development, ex vivo concentrations are based on the approved or tested doses. Regarding in vivo dosing, we would suggest using a moderately effective dose of the drug and then escalating to the maximal dose tolerable to the patient to obtain maximal benefits.

### Cell proliferation assays

For cell proliferation studies, 5,000 cells per well were plated in 96-well culture plates. After overnight incubation, the cells were treated with indicated concentrations of drug agents alone or in combination. Following a 48 hour incubation period, cellular proliferation was assessed using a tetrazolium dye reduction assay (CellTiter 96 Aqueous Non-Radioactive Cell Proliferation Assay; Promega, Madison, WI, USA) according to the manufacturer’s instructions. Absorbance was recorded on a microplate reader at 495 nm. Cellular proliferation was expressed as a percentage of vehicle-treated cells, which was defined as 100% viable.

### Western blotting

For the detection of various proteins, treated cells were lysed in lysis buffer (20 mM Tris, pH 7.4; 250 mM NaCl; 2 mM EDTA, pH 8.0; 0.1% TritonX-100; 0.01 mg/ml aprotinin; 0.005 mg/ml leupeptin; 0.4 mM PMSF; 4 mM NaVO4). Lysates were centrifuged at 14,000 rpm for 10 min to remove insoluble material and resolved on a 7.5% SDS gel. After electrophoresis, the proteins were electrotransferred to a nitrocellulose membrane, blocked with 5% non-fat milk, and probed with anti-cleaved PARP, Bcl2, AKT, phospho-AKT and NFκβ antibodies (1:1000 dilution; Cell Signaling Technology, Danvers, MA, USA) overnight at 4°C. An anti-β-actin antibody (Sigma, St. Louis, MO, USA) was used as a loading control. The blot was washed, exposed to HRP-conjugated secondary antibodies (Cell Signaling Technology, Danvers, MA, USA) for 1 hour, and examined by chemiluminescence (ECL; GE Healthcare, Little Chalfont, Buckinghamshire, UK).

## Results and discussion

Here, we present 4 case studies wherein personalized therapies were designed for different MM patient profiles that were then validated *ex vivo* using patient cells.

### Case study 1

**Figure 2:**
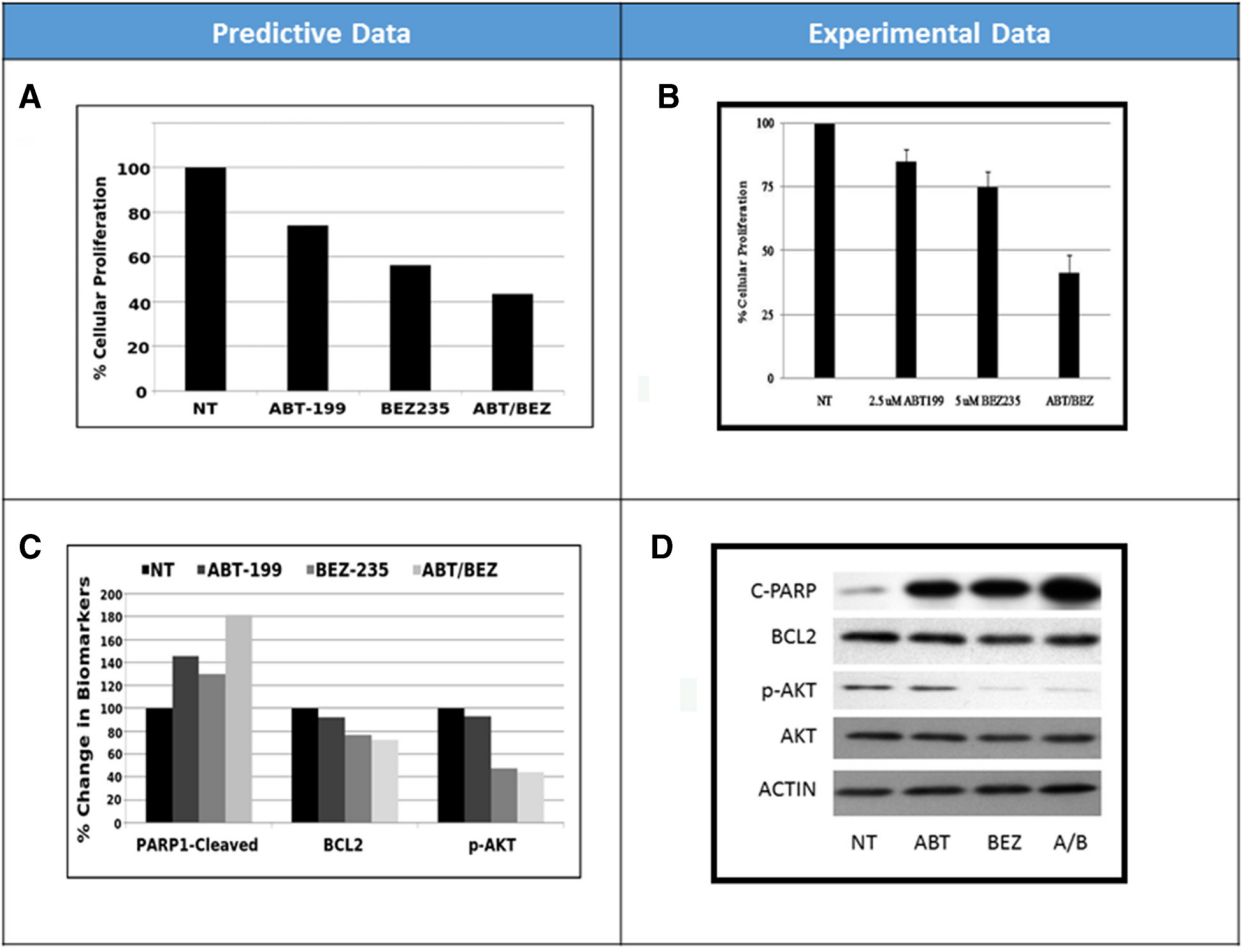
**MM patient 1 predictive vs. experimental results.** Predictive **(A)** vs. experimental **(B)** results regarding the effect of ABT199 and BEZ235 alone and in combination on cell proliferation in MM patient 1. Predictive **(C)** vs. experimental **(D)** results regarding the effect of ABT199 and BEZ235 alone and in combination on the biomarkers cleaved PARP1, BCL2 and AKT1.
- Discussion
  The patient-specific therapeutic simulation model was conceptually deduced using distinctive information from tumor signature as presented in Figure 3. The patient reported with chromosome 13 monosomy that included heterozygous deletion of the tumor suppressor genes RB1 and FOXO1 and homozygous deletion of the P53 gene, thereby presenting with high levels of BCL2, reduced PTEN and IGFBP3 levels, and a consequential increase in AKT levels. Inhibiting the PI3K/mTOR axis along with a BCL2 mimetic resulted in a reduction of cell proliferation and apoptosis biomarkers (AKT and BCL2). The combination synergistically reduced cell viability and proliferation and demonstrated convergence at increased apoptotic biomarkers, such as increased cleaved PARP1.

- Patient genomic characteristics and creation of patient simulation avatar
  FISH studies revealed loss of signals of chromosome 13 as well as a reported loss of chromosome 17p13.1, which corresponds to loss of the P53 gene. The genes present on the chromosome 13 were mapped, and 19 genes out of a total of 875 present in the Cellworks simulation model were perturbed (knock-down) along with a deletion of the P53 gene; these genes were used to create the patient simulation avatar.
- Design of individualized therapy
  Upon analysis of the patient characteristics, this profile with chromosome 13 monosomy and P53 deletion exhibited increased BCL2 levels and reduced PTEN and IGFBP3 levels, which are downstream of P53. In addition, a consequential increase in AKT was noted. Our analyses predicted a combination of BEZ235 (PI3K/mTOR inhibitor) and ABT-199 (BCL2 mimetic) for a synergistic reduction in the patient’s primary cell viability and proliferation phenotypes. The combination therapy was predicted to reduce key biomarkers, such as AKT and BCL2, and impact the downstream activation of pro-apoptotic markers, such as PARP1 and caspases.
- *Ex vivo* validation
  Given the personalized therapy prediction for MM patient 1, the inhibition of the PI3K/mTOR axis along with BCL2 was predicted to cause a synergistic reduction in cell growth and viability, and the effect is predicted to be significantly increased compared with the individual drugs. These predictive results were prospectively validated in patient cells *ex vivo*, and a synergistic reduction in cellular proliferation was observed experimentally (Figure 2A and B). ABT-199 (2.5 μM) only reduced proliferation by approximately 15%, whereas 5 μM BEZ235 reduced proliferation by 25%. In combination at the same concentrations, the reduction in cellular proliferation is synergistic with a 60% reduction (Figure 2B). The phosphorylated AKT biomarker exhibited enhanced growth reduction with the PI3K/mTOR inhibitor BEZ2355, and the same trend was observed upon combination treatment (Figure 2D). The reduction in BCL2 expression was not greater with the BCL2 mimetic since the agent only competes with BCL2 for the sequestration of the pro-apoptotic proteins. A reduction in BCL2 expression could potentially occur with NFkβ inhibition via reduction in phosphorylated AKT (Figure 2C and D). A synergistic increase in the pro-apoptotic biomarker cleaved PARP1 was observed with the combination, which correlated with the predictive trend for PARP1 (Figure 2C and D). Due to reduction of the dominant AKT pathway and BCL2 activity with the two inhibitors, there is a convergence downstream in the activation of apoptosis from these two different pathways as indicated by the increase in PARP1.

**Figure 3:**
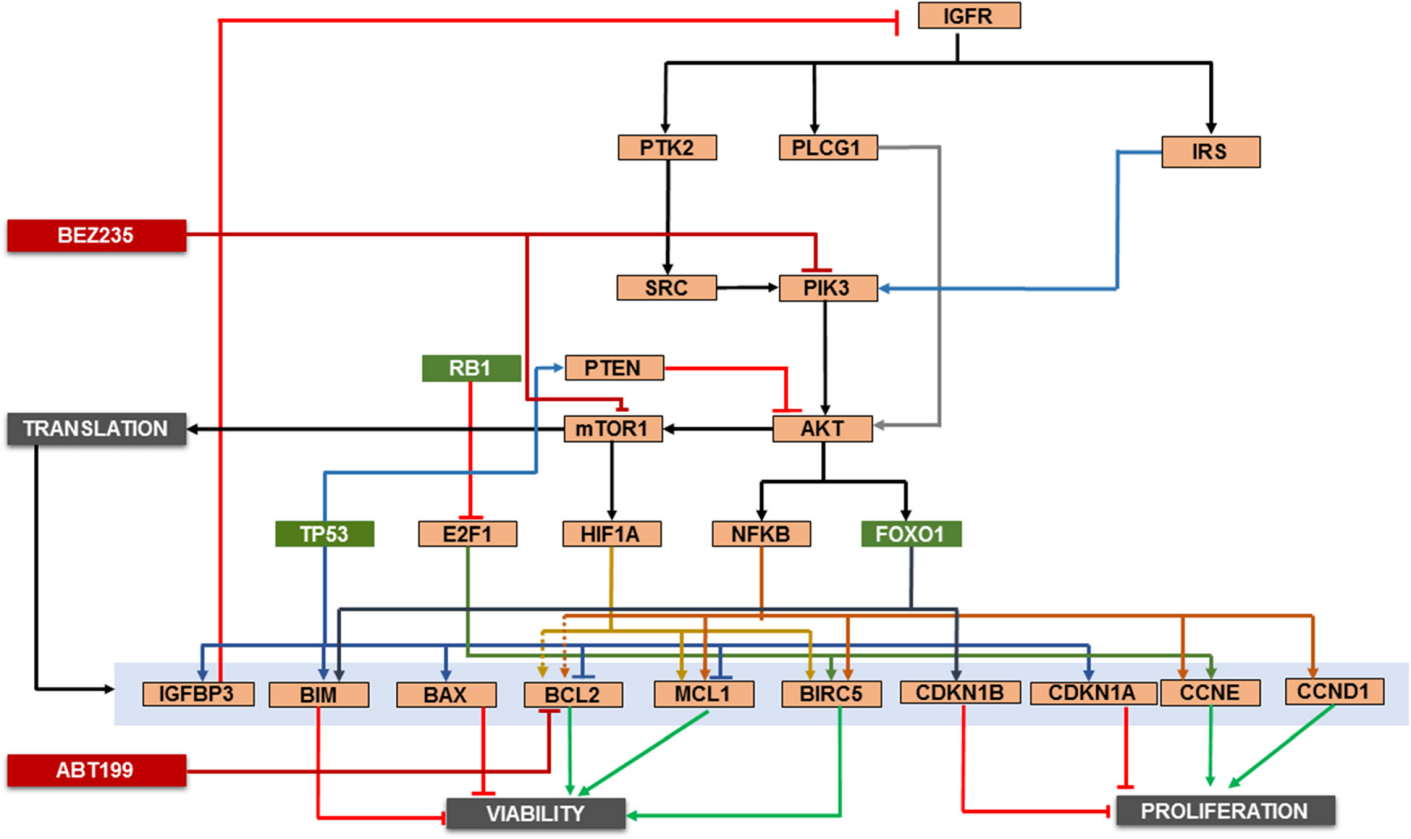
**MM patient 1 profile schematic.** The key signaling pathways and crosstalk relevant for MM patient 1 are presented. The profile includes chromosome 13 monosomy, loss of chromosome 17p13.1, heterozygous deletions of tumor suppressors RB1 and FOXO1 and a homozygous deletion of P53. Genes presented in green exhibit reduced expression. This profile displayed high AKT activation due to reduced PTEN and IGFBP3 expression, which are downstream of P53. Also, the simulation avatar predicted high BCL2 levels. The combination of BEZ235 (PI3K/mTOR inhibitor) and ABT199 (BCL2 mimetic) was predicted to result in a synergistic reduction in proliferation and viability endpoints.

### Case study 2

**Figure 4:**
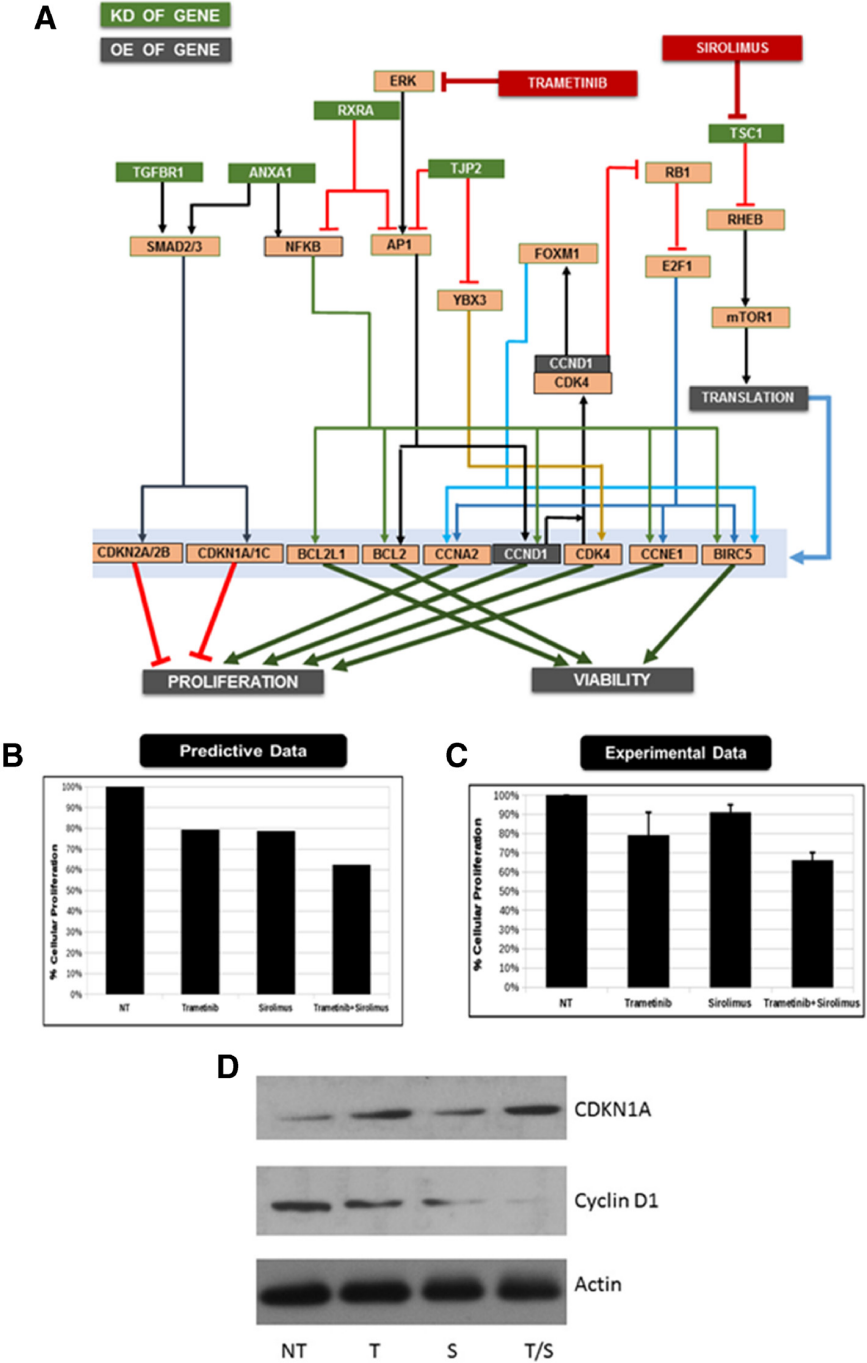
**MM patient 2 profile schematic. (A)** The key signaling pathways and crosstalk relevant for MM patient 1 are presented. This profile exhibits a chromosome 11:14 translocation, losses in chromosomes X and 9, amplification of CCND1 and a knockdown of tumor suppressors RXRA, TGFBR1, TJP2 and TSC1. The combination of trametinib (ERK inhibitor) and sirolimus (mTOR1 inhibitor) were predicted to demonstrate optimal efficacy. The predictive **(B)** vs. experimental **(C)** results regarding the impact of sirolimus and trametinib individually and in combination on relative cell growth. **(D)** Western blot of non-treated (NT), trametinib (T), sirolimus (S) and combination (S/T) for the biomarker cyclin D1 and CDKN1A. Actin serves as a loading control.
- *Ex vivo* validation
  Sirolimus and trametinib were assessed individually and in combination *ex vivo* on the patient cells. Sirolimus (5 μM) exhibited a 10% decrease in cellular proliferation, whereas 5 μM trametinib caused an approximate 20% decrease. Combination of the two drugs at the same concentration caused a 35% decrease in cellular proliferation, thus confirming the prediction (Figure 4B and C). In addition, the combination increases CDKN1A expression and reduces cyclin D1 expression (Figure 4D).
- Discussion
  As demonstrated in the signaling network of the key characteristics of the patient cancer cells illustrated in Figure 4A, sirolimus inhibits the aberrantly activated mTOR1 signaling due to TSC1 knockdown, whereas trametinib significantly impacts AP1 activation via ERK inhibition. These effects converge by reducing the amplified cyclin D1 and other key proliferative genes.

- Patient genomic characteristics and creation of patient simulation avatar
  The aCGH and FISH analysis of the bone marrow sample included losses in chromosomes X and 9 as well as a chromosome 11:14 translocation that is a commonly reported in MM. This translocation results in increased CCND1 expression. The genomic aberrations reported included knockdown of tumor suppressors RXRA, TGFBR1, TJP2 and TSC1.
- Design of individualized therapy
  The simulation avatar of this patient profile revealed increased mTOR signaling. TSC1 negatively regulates mTOR1 pathway, and its deletion in the patient’s genomic profile is predicted to cause aberrant activation of mTOR1 and the pathway’s downstream targets. A significant increase in the proliferation endpoint is attributed to amplification of cyclin D1 expression, increased activation of AP1 and NFkβ due to reduced RXRA and TJP2 expression and a reduction in cell cycle inhibitors that are regulated by TGFb-mediated SMAD signaling. Modeling predicted a combination of the mTOR1 inhibitor sirolimus and the ERK inhibitor trametinib to be efficacious for this profile (Figure 4A).

### Case study 3

**Figure 5:**
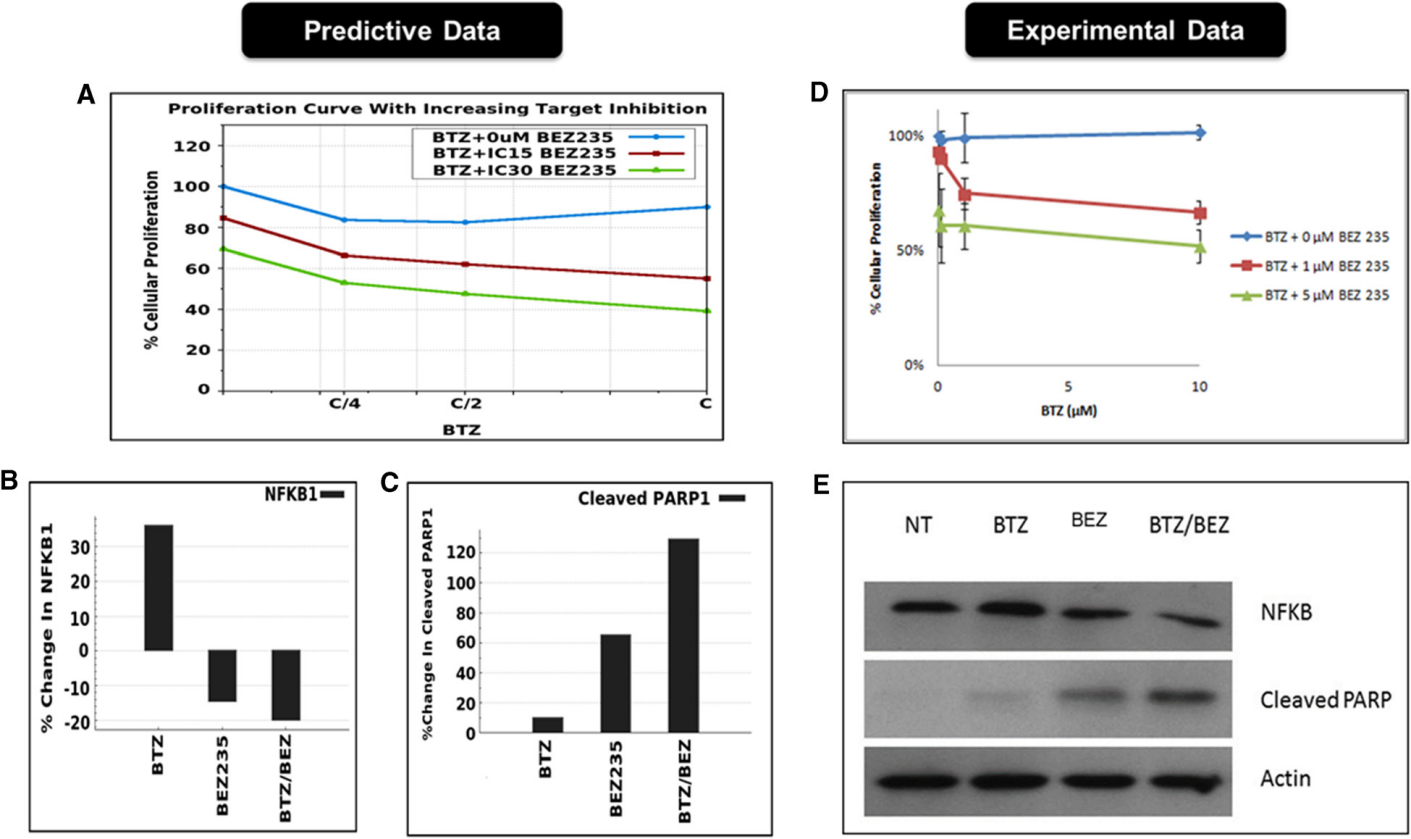
**MM patient 3 predictive vs. experimental results.** Predictive **(A)** vs. experimental **(B)** results regarding the effect of bortezomib and BEZ235 alone and in combination on cell proliferation in MM patient 3. Increasing doses of bortezomib are plotted on the x-axis, represented as fractions of concentration C (~70% target inhibition) for the predictive plot **(A)** and as micromolar concentrations of the drug in the experimental plot **(B)**. The % change in proliferation from untreated baseline is plotted on the y-axis. Predictive results for the effect of bortezomib and BEZ235 alone and in combination on NFkβ **(C)** and cleaved-PARP1 **(D)**, with the % change in the biomarker from untreated baseline plotted on the y-axis. **(E)** Western blot of non-treated (NT), bortezomib (BTZ) alone, BEZ235 (BEZ) alone and the combination (BTZ/BEZ) on the biomarkers NFkβ and cleaved PARP1. Actin serves as the loading control.
- Discussion
  The key characteristics of the patient simulation avatar included CUL1, CAV1 deletion, translocation of chromosome 11/14 and resulting amplification of CCND1 and AKT1 (Figure 6). CUL1 is the ubiquitin E3 ligase that facilitates targeting of many proteins, including Notch, CTNNB1, and NFkβIA, for proteasomal degradation [26]. A CUL1 deletion indicates a defect in this targeting and therefore protein accumulation. Bortezomib is an inhibitor of the proteasome, and the therapeutic effect of proteasome inhibition is mediated via accumulation of the tumor suppressor proteins, such as cell cycle inhibitors that aid in controlling increased tumor cell proliferation [27]. In this profile, an accumulation of the tumor promoter genes is expected based on CUL1 and CAV1 deletions, and further inhibition of the proteasome with bortezomib is predicted to further increase the expression of these tumor promoters. Thus, no reduction in the tumor endpoints is expected, resulting in drug resistance. AKT1 is overexpressed due to 11/14 chromosome translocation. The PI3K-AKT pathway is more dominant than the ERK pathway in this profile. The individualized therapy was designed to target the PI3K/mTOR axis to overcome bortezomib resistance. The addition of the PI3K/mTOR inhibitor BEZ235 potentially causes reduced AKT expression and impacts global translation machinery via mTOR inhibition. This allows bortezomib–mediated proteasome inhibition to become more effective by tilting the balance towards the tumor suppressor accumulation over the effect of oncogene accumulation, thereby making this combination effective in this profile as confirmed by the *ex vivo* studies on the patient cells.

- Patient genomic characteristics and creation of patient simulation avatar
  A deletion of chromosome 7 from region q22 to q36, Del(7)(q22q36) that contained the CAV1, BRAF, CUX1, EZH2, MET, NRF1, SMO, CUL1 and RHEB genes was noted from the aCGH and SKY profiling of the patient tumor. This patient profile also contained the commonly reported MM translocation t(11; 14)(q13; q32) that results in an amplification of the following genes: AIP, PDEA2, TCL1A, RCE1, CCND1, MAP4K2, MAP3K11, AKT1, YY1, FOSL1, FGF19 and IL18BP. The deletion and amplification of the above genes was input to create the patient simulation avatar. Loss of CUL1 due to deletion of chromosome 7q36.1 region and the gain of CCND1 as a result of gain of chromosome 11q13 region were identified as key patient characteristics.
- Design of individualized therapy
  The predictive results indicate bortezomib resistance in this profile. Based on the dose response studies, IC15 and IC30 viability was determined for the responsive drugs, which were combined at these concentrations with bortezomib in an exhaustive combination simulation experiment. We then shortlisted combinations that exhibited synergistic efficacy on the tumor endpoints by reducing viability and proliferation in combination with bortezomib. The combination of bortezomib with BEZ235 (PI3K/mTOR inhibitor) aligned the best with the driver pathways in the patient profile and was prospectively validated *ex vivo* in patient-derived tumor cells.
- *Ex vivo* validation
  A bortezomib dose response was performed individually and in combination with 1 and 5 μM BEZ235 (Figure 5B). Consistent with the predictions, this profile was resistant to increasing doses of bortezomib, and this resistance was overcome with the addition of BEZ235 (Figure 5A). Predictions of increased NFkβ in the basal profile with bortezomib (Figure 5C) were validated in the *ex vivo* sample (Figure 5E), and the combination revealed an enhanced decrease in NFkβ and a synergistic increase in the apoptotic marker cleaved PARP1 (Figure 5D and E).

**Figure 6:**
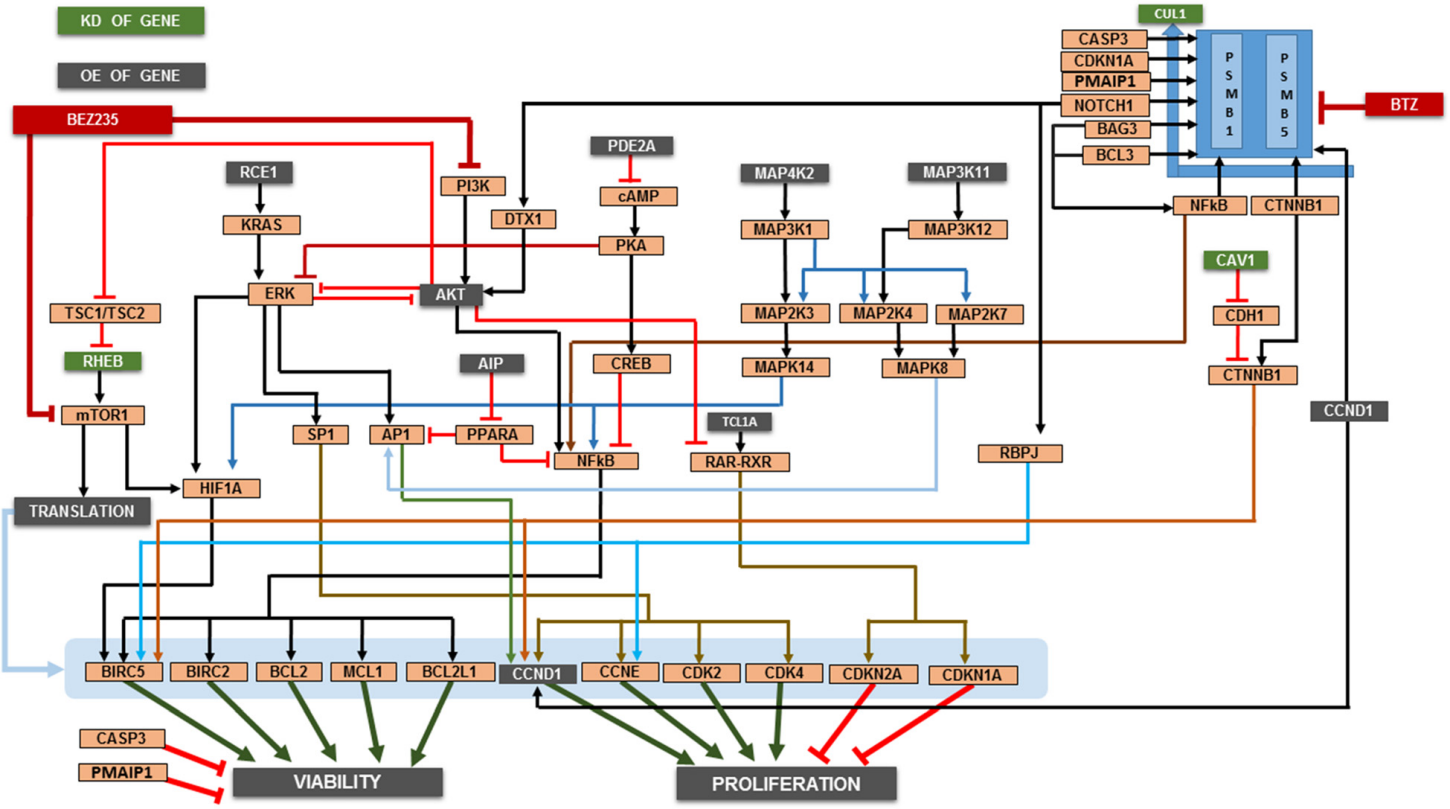
**MM patient 3 profile schematic.** The key signaling pathways and crosstalk relevant for MM patient 3 are presented. This profile contained multiple aberrations, including a chromosome 11:14 translocation. The profile exhibited a CUL1 deletion and increased CTNNB1; the patient was clinically resistant to bortezomib. The profile also contained an amplification of RHEB1, implicating activation of the mTOR pathway. The PI3K/mTOR inhibitor BEZ235 in combination with bortezomib was predicted to be effective in this patient.

### Case study 4

**Figure 7:**
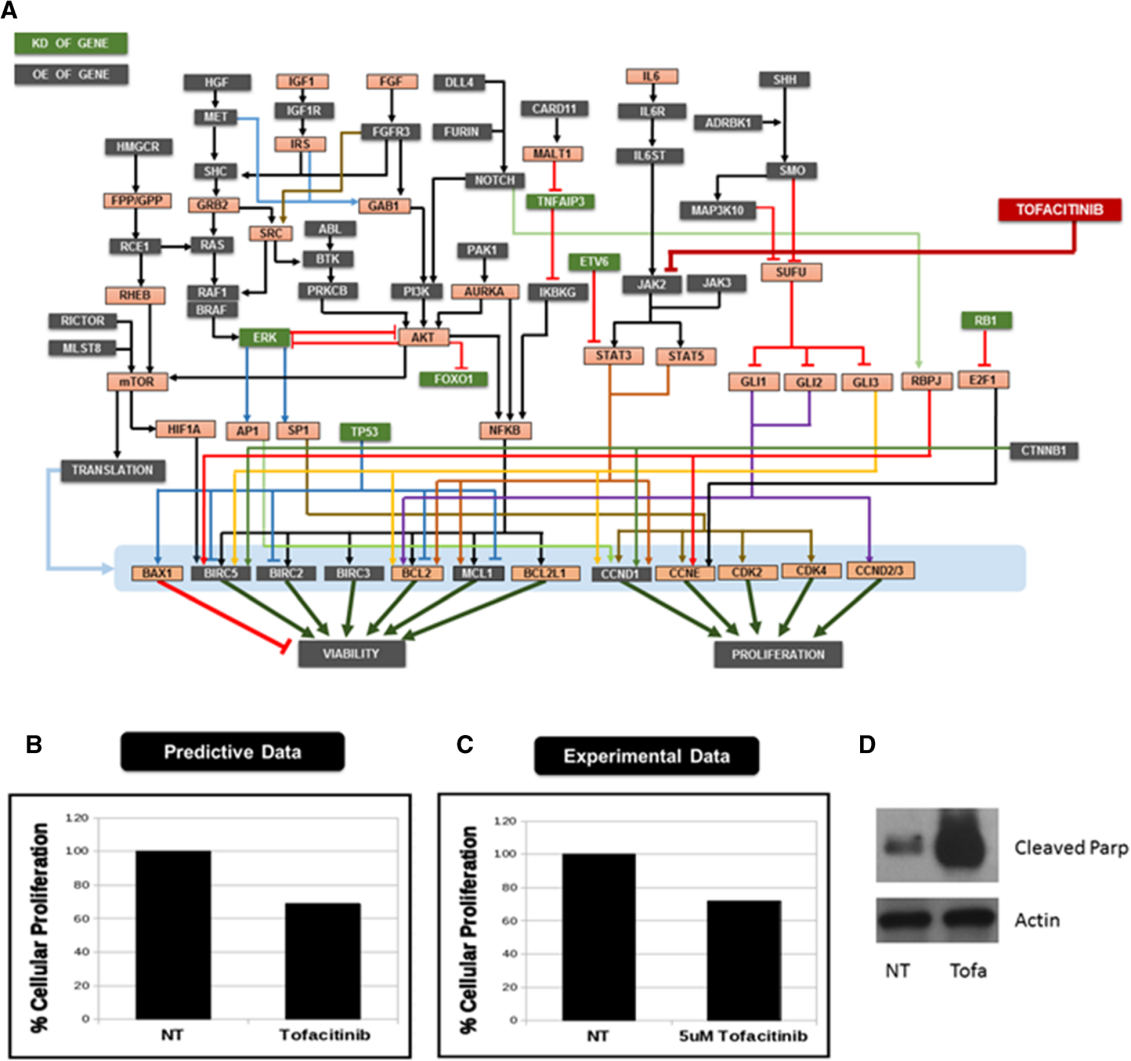
**Data for MM patient 4. (A)** Rationale for MM patient 4 patient characteristics and predicted effects on the network. This profile exhibited CCND1 trisomy, P53 deletion and extensive aberrations and hyperdiploid clones. The simulation avatar exhibited significant activation of the ERK, AKT and JAK-STAT signaling pathways. The JAK inhibitor tofacitinib, represented in the red box, was predicted to be effective in this profile. Predictive **(B)** vs. experimental **(C)** results regarding the effect of tofacitinib on cell proliferation in MM patient 4. **(D)** Western blot of non-treated (NT) and Tofacitinib (Tofa)-treated cells. The biomarker cleaved PARP was assessed. Actin serves as a loading control.
- *Ex vivo* validation
  Figure 7B and C demonstrate a tight correlation between the predictive and experimental results. An approximate 30% reduction in cell growth was noted upon treatment with 5 μM tofacitinib. A similar trend is noted in the predictive plot. Furthermore, treatment with 5 μM tofacitinib induces PARP cleavage.
- Discussion
  This profile exhibited a large number of aberrations, many of which appeared to be significant drivers based on their contributions in other cancers, including the growth factors MET, IGFR, FGFR and Hedgehog and NOTCH signaling pathways. However, simulation analysis shortlisted the JAK inhibitor for this profile due to the significant activation of STAT3 and STAT5 in the patient simulation avatar. This was attributed to the high CNV of the IL6 receptor, the amplification of IL6 pathway and high CNV of JAK2 and JAK3, which are downstream the IL6 pathway. Thus, this pathway was predicted to be a key driver in this profile. A combination of the JAK inhibitor with other modulators that target the other highly up-regulated pathway could possibly exhibit increased efficacy; however, due to the lack of availability of additional patient cells, we could only validate and confirm the efficacy of tofacitinib and the dominance of the JAK-STAT signaling in this profile.

- Patient genomic characteristics and creation of patient simulation avatar
  Input for patient profile creation included karyotype data and FISH analysis. Based on the cytogenetics and clinical genomics report, patient aberrations included trisomy of CCND1 and deletion of P53 along with single copy losses in different arms of chromosomes 1, 6, 8, 12, 13, 14, 16, 17 and 22 as well as gains in different arms and regions of chromosomes X, 1, 4, 7, 9, 17, 3, 5, 11, 15, and 19, indicating the presence of hyperdiploid clones. Using this information, 897 gene perturbations were used to model this patient simulation avatar.
- Design of individualized therapy
  The key characteristics of this profile are presented as a network schematic (Figure 7A). Simulation predicted increased beta-catenin (CTNNB1) activity with increased hedgehog and NOTCH pathways. The hedgehog pathway was amplified due to high CNV of SHH, SMO and ADBRK1. The profile exhibited high CNV of DLL4, FURIN and NOTCH1, thereby increasing NOTCH signaling. Significant activation of STAT3 and STAT5 were noted in the simulation avatar due to amplification of the IL6 pathway as well as JAK2 and JAK3. High CNV of MET, IGFR and FGFR converged at the ERK and AKT signaling loops. Along with deletion of P53, this profile exhibited amplification of many anti-apoptotic genes including, survivin, MCL1 and XIAP. High throughput simulation-based testing of the drug library on the patient simulation avatar identified the JAK inhibitor tofacitinib, a drug approved for rheumatoid arthritis, to be effective for this profile.

## Conclusion

Drug resistance and myeloma relapse are commonly observed in MM patients [2]. Using clinical trials to develop alternative drug therapies for MM patients (resistant to a particular therapy) typically requires several years of validation and risk assessment [28]. We developed and validated a predictive patient specific simulation model that can design therapeutics in a shorter time period with potentially increased success.

Rather than the conventional one gene mutation influencing the sensitivity of a drug approach (i.e., recommendation of use of an EGFR inhibitor in an EGFR over-expressed or mutant profile), our personalized therapy regime is based on the impact of drugs on the patient’s signaling networks driven by multiple chromosomal aberrations. Our personalized therapy design requires simulation-based analyses of the dominance of key biomarkers, pathways and convergence points within the patient network.

The predictive simulation approach comprehensively models signal transduction, epigenetic regulation, regulation of protein homeostasis (proteasome and autophagy), metabolic pathways and other regulations representing all cancer phenotypes with ongoing enhancements. The technology has the ability to predict clinical outcomes and enables the design of personalized therapeutic assets and novel biomarkers for stratification of target patient sub-groups.

The therapeutic strategy uses repurposing of molecularly targeted drug agents from across indications to shortlist therapeutic combinations. The repurposed drug agent list includes those that are clinically safe but were ultimately not approved due to an inability to achieved efficacy goals, sunset FDA-approved agents for use at end of patent life and other FDA approved on-patent and off-patent generic agents. The recycling and reuse of approved and clinically tested drugs provides a faster path to clinical translation [29].

The simulation technology and approach is highly differentiated from other pathway analysis approaches due to its simulation capability. The simulation predictions have been prospectively validated [18]. Novel and non-obvious predictions have created a pipeline of biomarker and therapeutic patented assets.

Various limitations to this study should be noted. First, we are unable to separate potential clones from the patient sample. Therefore, we are unable to determine whether each drug in the personalized drug combination is selectively targeting individual patient clones or acting collectively. Some of the drugs produce weak inhibition (less than 20% growth inhibition), which may not be clinically relevant. In addition, CD138+ clones were not selected for in a number of the cases (Cases 1-3). Thus, the cells used to validate the predictive findings ex vivo may not be completely representative of the patient’s tumor cells.

Intra-tumor heterogeneity is a significant issue that plagues the development of effective treatments. Although not specifically addresses in this study, intra-tumor heterogeneity could be potentially addressed by this system using profiling data on multiple different clones from the same patient. Data from these clones could be individually modeled to identify a treatment option that would be effective in all clones. In addition, this platform could also be utilized to explore differential gene expression at different stages of tumor progression and predict corresponding drug regimens. If profiling information of the tumor sample at different stages or time points in disease progression could be obtained, each sample could be modeled individually. By comparatively analyzing avatars from the various stages, one could obtain insight into the appearance of new aberrations and changes that potentially influence disease progression. Treatment alterations could be made as the disease progresses if this information was available.

Moreover, future iterations of our platform could provide enhance predictive data. Currently, the dataset used to create the patient simulation avatar is based on copy number variation and mutation data. Proteomics data could potentially be used to confirm the differential expression of proteins in the patient network based on the genomic profile and would provide increased confidence in the patient avatar predictions. Metabolomics data set would also provide an additional patient avatar validation and alignment dataset, thus strengthening the prediction model. Although we are currently unable to include this information in our platform, future studies involve enhancing our platform’s capacity to predict effective drug combinations based on genetic, proteomic, and metabolomic data.

One challenge in using this information would involve closely monitoring the side effects of novel drug combinations. Most of the drugs in the library are currently marketed with known safety and toxicity profiles and have been administered for extended durations in humans. However, the effects of drugs in combination are unknown. The safety of these combinations could be assessed in animal models before being administered in a clinical setting.

The design of personalized therapy based on this approach has the added advantage of addressing resistance mechanisms upfront that can occur when therapy is designed based on presence of single mutations. One mutation or perturbation does not take into account the other aberrations that can contribute to resistance. For example, in case study 3, the presence of a deletion in CUL1 could be responsible for making the profile less sensitive to bortezomib. In addition, the presence of other alterations, such as low CNV of APC or CDH1 or mutations in the RAF-ERK-AP1 loop that has inherently high levels of beta-catenin (CTNNB1) in the base profile, could also contribute to rapid development of bortezomib resistance in the profile. These potentially useful clinical insights were obtained via this simulation approach. Our promising data warrant validation in future clinical trials to advance the personalized care of MM patients.

## Additional file

Additional file 1:
**Appendix: Abbreviations used in the patient profile schematics.**
