# Supplementary material for: Personalization of cancer treatment using predictive simulation
Source: J Transl Med. 2015 Feb 1;13:43. doi: 10.1186/s12967-015-0399-y (PMC4320499; doi:10.1186/s12967-015-0399-y)
Supplement: Additional file 1: — Appendix: Abbreviations used in the patient profile schematics. [file 12967_2015_399_MOESM1_ESM.docx]

**Appendix: Abbreviations used in the patient profile schematics**

ADBRK1/ADRBK1 - Adrenergic, beta, receptor kinase 1

AIP - Aryl hydrocarbon receptor interacting protein

AKT - V-AKT murine thymoma viral oncogene homolog 1

AP1 - Activated protein 1, complex of Fos and Jun

APC - Adenomatous polyposis coli

BCL2 - B-cell CLL/lymphoma 2

BIRC3 - Baculoviral IAP repeat containing 3

BRAF - B-Raf proto-oncogene, serine/threonine kinase

CAV1 - Caveolin 1

CCL5 - Chemokine (C-C motif) ligand 5

CCND1 - Cyclin D1

CD138 - Syndecan 1

CDH1 - Cadherin 1, type 1, E-cadherin

CDK - Cyclin-dependent kinases

CDKN1A - Cyclin-dependent kinase inhibitor 1A (p21, Cip1)

CTNNB1 - Catenin (cadherin-associated protein), beta 1, 88kDa

CUL1 - Cullin 1

CUX1 - Cut-like homeobox 1

DLL4 - Delta-like 4

EGFR - Epidermal growth factor receptor

ENSEMBL - Genome database

ERK - Mitogen-activated protein kinase 1

EZH2 - Enhancer of zeste 2 polycomb repressive complex 2 subunit

FGF19 - Fibroblast growth factor 19

FGFR - Fibroblast growth factor receptor

FOSL1 - FOS-like antigen 1

FOXO1 - forkhead box O1

FURIN - Furin (paired basic amino acid cleaving enzyme)

IGFBP3 - Insulin-like growth factor binding protein 3

IGFR - Insulin-like growth factor receptor

IL6 - Interleukin 6

IL18BP - Interleukin 18 binding protein

JAK2 - Janus kinase 2

JAK3 - Janus kinase 3

MAP3K11 - Mitogen-activated protein kinase kinase kinase 11

MAP4K2 - Mitogen-activated protein kinase kinase kinase kinase 2

MCL1 - Myeloid cell leukemia 1

MET - MET proto-oncogene, receptor tyrosine kinase

mTOR - Mechanistic target of rapamycin (serine/threonine kinase)

NfkB - Nuclear factor of kappa light polypeptide gene enhancer in B-cells 1

NfkBIA - Nuclear factor of kappa light polypeptide gene enhancer in B-cells inhibitor, alpha

NOTCH1 - Notch 1

NRF1 - Nuclear respiratory factor 1

P53 - Tumor protein p53

PARP1 - Poly (ADP-ribose) polymerase 1

PDEA2/PDE2A - Phosphodiesterase 2A, cGMP-stimulated

PI3K - Phosphatidylinositol-4,5-bisphosphate 3-kinase, catalytic subunit

PTEN - Phosphatase and tensin homolog

RB1 - Retinoblastoma 1

RCE1 - Ras converting CAAX endopeptidase 1

RHEB - Ras homolog enriched in brain

RXRA - Retinoid X receptor, alpha

SHH - Sonic hedgehog

SMAD - SMAD family member

SMO - Smoothened, frizzled class receptor

STAT3 - Signal transducer and activator of transcription 3

STAT5 - Signal transducer and activator of transcription 5

TCL1A - T-cell leukemia/lymphoma 1A

TGFb - Transforming growth factor, beta 1

TGFBR1 -Transforming growth factor, beta receptor 1

TJP2 - Tight junction protein 2

TSC1 - Tuberous sclerosis 1

WNT - Wingless-type MMTV integration site family member

XIAP - X-linked inhibitor of apoptosis, E3 ubiquitin protein ligase

YY1 - YY1 transcription factor
